# Effects of* Streptococcus sanguinis* Bacteriocin on Deformation, Adhesion Ability, and Young's Modulus of* Candida albicans*

**DOI:** 10.1155/2017/5291486

**Published:** 2017-05-22

**Authors:** Shengli Ma, Wenyu Ge, Yifan Yan, Xu Huang, Li Ma, Chunmei Li, Shuyang Yu, Chunxiao Chen

**Affiliations:** ^1^Department of Stomatology, Hospital of Heilongjiang Province, Harbin 150036, China; ^2^University of Jiamusi, Jiamusi 154002, China

## Abstract

In order to study the thallus changes on microscopic morphology and mechanical properties of* Candida albicans* antagonized by* Streptococcus sanguinis* bacteriocin, the adhesion ability and Young's modulus of thalli and hypha of* Candida albicans* were measured by the relative measurement method using atomic force microscope's (AFM) tapping model. The results showed that the average adhesion ability and Young's modulus of thalli were 7.35 ± 0.77 nN and 7.33 ± 1.29 Mpa, respectively; the average adhesion ability and Young's modulus of hypha were 9.82 ± 0.39 nN and 4.04 ± 0.76 Mpa, respectively. After being antagonized by* Streptococcus sanguinis* bacteriocin, the adhesion ability was decreased along with the increasing of deformation in reaction region and Young's modulus followed the same changes. It could be concluded that the adhesion ability of hypha was greater than thalli, Young's modulus of hypha was less than thalli, and adhesion ability and Young's modulus of* Candida albicans *were decreased significantly after being antagonized by* Streptococcus sanguinis* bacteriocin.

## 1. Introduction

Fungal infection became a tremendous threat to human health; fatality rate of deep fungal infection was higher than 80% [1], so it was an urgent problem to be solved out.* Candida albicans (C. albicans)* was quite common in human fungal infection; it could encroach on human skin, mucosa, and internal organs [2]. As the widespread application of immunosuppressive agents, broad spectrum antibiotics, and antitumor drugs, incidence rate of deep fungal infection was increased every year [3, 4].* Streptococcus sanguinis (S. sanguinis) *was a dominant bacteria in healthy human oral cavity; it was reported that* S. sanguinis* had a strong antagonistic effect on* C. albicans *[5]. An antibacterial substance generated by* S. sanguinis* was called* S. sanguinis *bacteriocin* (S. s bacteriocin)*; it was reported that morphology of* C. albicans* was altered after treatment with* S. s bacteriocin*, resulting in disc-like depressions in the surfaces of the spores and mycelia [6].* S. s bacteriocin* could even change the cell membrane permeability of* C. albicans*, resulting in the leakages of cell contents [7, 8]. However, the mechanism of the inhibitory effect of* S. s bacteriocin* had not been reported yet. It had been proved that cells had mechanical character to respond to physical and chemical stimulus from outside and make autoregulation, such as Young's modulus and adhesion ability [9]. From the changes of cytomechanical character, we can suspect the cellular changes in the physiological and pathological processes [10–12]. In this study, effects of* S. s bacteriocin* on adhesion ability and Young's modulus of* C. albicans *were analyzed, in order to reveal the mechanical effects of* S. s bacteriocin* on fungi.

## 2. Materials and Methods

### 2.1. Extraction of* S. s bacteriocin*

The standard strain ATCC 10556 of* S. sanguinis* was purchased from the State Key Laboratory of Oral Diseases, West China College of Stomatology, Sichuan University (Chengdu, China). Following identification and pure culture, the bacteria were inoculated on Brain Heart Infusion (BHI) culture medium and anaerobically cultured for 48 h; the medium of* S. sanguinis* was ultracentrifuged at a low temperature following anaerobic culture. The bacterial precipitate was collected, washed, and resuspended in phosphate-buffered saline (PBS). The target bacteriocin was released from the cells by sonication and the supernatant was collected by centrifugation (12,000 ×g, 4°C, 30 min). Solid ammonium sulfate was slowly added to the supernatant to yield 60% saturation, salted out for 6 h at 4°C and centrifuged to isolate the supernatant; the precipitate was dissolved in PBS. The desalting purification was conducted by chromatography on Sephadex G-25 (Pharmacia, Picastaway, NJ, USA). The collected materials were dialyzed, condensed, lyophilized, and cryopreserved [13].

### 2.2. Culture of* C. albicans*

Standard strain ATCC10231 of* C. albicans* was purchased from Shanghai Fu Xiang Biotechnology Co. Ltd.; the purified and identified* C. albicans* was diluted to 5 × 10^6^ cfu/mL with RPMI-1640 culture medium; the bacterial suspensions were adjusted to PH 7.0 and cultured in 37°C for 2.5 h; after germ tube induction, they were centrifuged. The precipitate was collected, washed, and resuspended in PBS. Then 10 *μ*L suspensions were dropped on fresh peeled mica surface; after atmospheric drying, they were used for atomic force microscope (AFM) analysis [14].

### 2.3. Illustration of the AFM Methods

**Figure 1:**
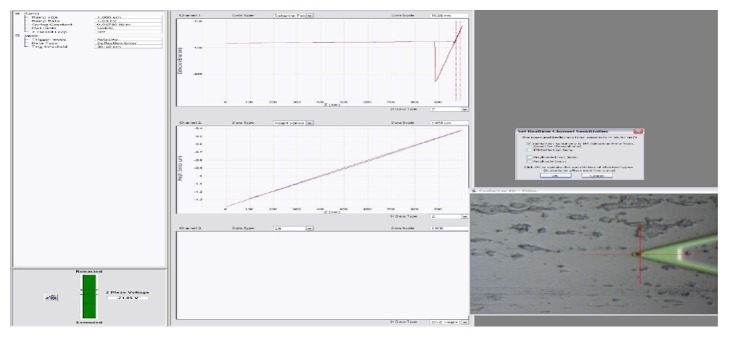
Force curves of* C. albicans* in single indentation point.

The adhesion force and Young's modulus of the cell were measured by real-time analyzing of the recorded force curves using AFM Peak Force QNM mode, under atmosphere; observation was carried out using standard scanning head with maximum sweep range of 100 *μ*m × 100 *μ*m × 15 *μ*m; passivating probe was selected to do indentation tests with viable cells and the force curves were recorded; elastic constant of commercialized AFM cantilever was marked on the probe box before leaving the factory, but these force constants were calculated based on the homogeneous cantilever, so there were some deviation with normal value; after recalibration, elastic modulus of Si3N4 probe was 0.06 N/m, resonant frequency of needlepoint was 17 kHz, the scanning speed was adjusted with the size of the scanning range, and frequency was controlled within 0.5–1.5 Hz. Dotty force curves were obtained quickly by scanning images while indenting samples using selected indentation frequency; after calculating the force curves, adhesion force and Young's modulus of each indentation point were obtained; the results were collected by morphology imaging and deflection imaging, image pixels were 256 × 256 [15, 16], and the original force curves for measuring these parameters were shown in Figure 1.

### 2.4. Measurements of Adhesion Ability and Young's Modulus of Normal* C. albicans *Thallus and Hypha

Using relative measurement, fiducial value was set to 3.5 Mpa, Hertz model was defined as cell model [16, 17], AFM planar imaging of* C. albicans *in budding period was selected, and cell center was the basal observation point; there were 4 survey lines chosen randomly by using “#” marked in the surface of thallus cells; the thallus was demarcated; XzDate value in each site of survey lines was measured, the XzDate value represented adhesion value in adhesion measurement channel, and it represented Young's modulus value in Young's modulus channel; the mean value was calculated; the mean values of 4 survey lines were summed and then averaged; finally, the accurate value of adhesion ability and Young's modulus was obtained, respectively. AFM planar imaging of* C. albicans* in budding period was selected randomly, gemmiparous hyphae with length of 8 to 20 *μ*m were selected and divided into 5 equal parts perpendicular to the 4 survey lines in long axis of hypha; XzDate value in each site of survey lines was measured. The hyphal calculating method of adhesion ability and Young's modulus was the same as thallus. Scanning time of each sample was within 2 h in this study and at least 8 cells were measured in each sample.

### 2.5. Measurements of Deformation, Adhesion Ability and Young's Modulus of* C. albicans* after* S. s bacteriocin* Treatment

The* S. s bacteriocin* was diluted to 1 g/L with PBS;* C. albicans* was diluted to 5 × 10^6^ cfu/mL with RPMI-1640 culture medium;* C. albicans* suspension (500 *μ*L) was inoculated into a culture tube with 1 mL RPMI-1640 and 200 *μ*L* S. s bacteriocin *for 24 h at 37°C and then prepared for smear examination. AFM planar imaging of* C. albicans* after* S. s bacteriocin *treatment was selected randomly; thalli were divided into 5 equal parts perpendicular to the 4 survey lines in long axis; XzDate value in each site of survey lines was measured in deformation channel, adhesion measurement channel, and Young's modulus channel, respectively. The mean values of 4 survey lines were summed and then averaged; finally, the accurate values of deformation, adhesion ability, and Young's modulus of* C. albicans* after* S. s bacteriocin* treatment were obtained, respectively.

### 2.6. Statistical Analysis

Data analysis was carried out using SPSS19.0 software; the measured data were represented by *x* ± *s* and analyzed with paired-samples *T* test; *p* < 0.05 was considered to have statistical significance.

## 3. Results

### 3.1. Adhesion Ability and Young's Modulus of Normal* C. albicans* Thallus and Hypha

**Figure 2:**
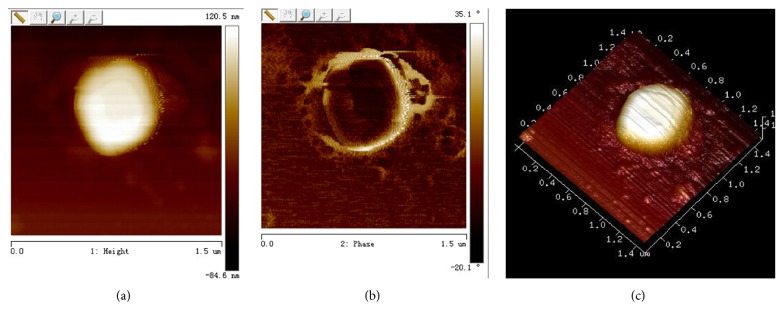
Single spores form of* C. albicans *measured by real-time analyzing of the recorded force curves using AFM Peak Force QNM mode. (a) Spores form of* C. albicans*, plane graph. (b) Spores form of* C. albicans*, phase graph. (c) Spores form of* C. albicans*, three-dimension graph.

**Figure 3:**
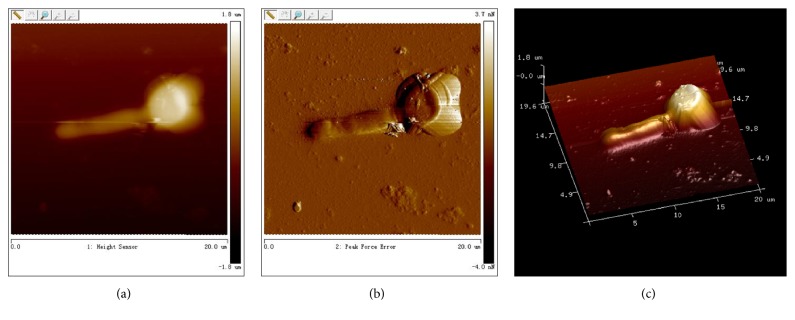
Single hyphal form of* C. albicans* measured by real-time analyzing of the recorded force curves using AFM Peak Force QNM mode. (a) Hyphal form of* C. albicans*, plane graph. (b) Hyphal form of* C. albicans*, phase graph. (c) Hyphal form of* C. albicans*, three-dimension graph.

Yeast form of* C. albicans *thallus was round or ellipse with smooth and flat surface (Figures 2(a)–2(c)), after gemmation induction; the length of hyphae ranged from 8 to 20 *μ*m; their surface was smooth and flat; there was no fracture or depression (Figures 3(a)–3(c)). In the range of 0~10.2 *μ*m, adhesion parameter of* C. albicans* hypha was 9~12 nN, while in the range of 10.2~16 *μ*m, the parameter was 7~10 nN and the value of adhesion ability of hypha was higher than thallus (Figure 5(b)). Take the site of 12.4 *μ*m in Figure 5(b) as reference point (adhesion ability was 0 nN); the adhesion ability was measured, the mean value of hypha was 9.82 ± 0.39 nN, and the thallus was 7.35  ±  0.77 nN (Table 1). Young's modulus of thallus was 7~9 MPa, while the hypha was 4~6 MPa, less than the thallus (Figure 5(c)). After statistical analysis, the mean value of Young's modulus of thallus was 7.33 ± 1.29 Mpa, and the hypha was 4.04 ± 0.76 Mpa (Table 2). It could be concluded that the adhesion ability of hypha was greater than thalli and Young's modulus of hypha was less than thalli.

### 3.2. Deformation, Adhesion Ability and Young's Modulus of* C. albicans* after* S. s bacteriocin* Treatment

**Figure 4:**
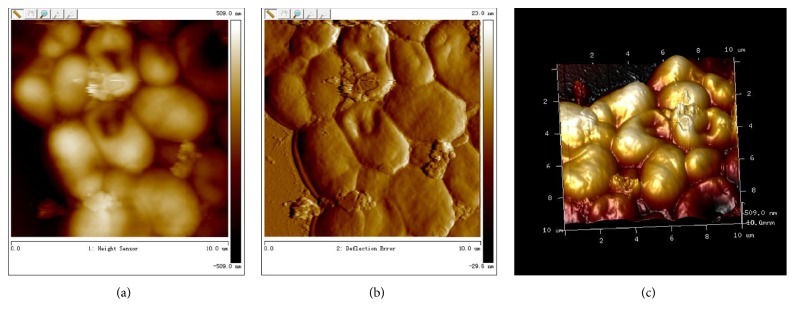
The imaging of groups of* C. albicans* antagonized by* S. s bacteriocin*. (a) The imaging of* C. albicans* antagonized by* S. s bacteriocin*, plane graph; (b) The imaging of* C. albicans* antagonized by* S. s bacteriocin*, phase graph; (c) The imaging of* C. albicans* antagonized by* S. s bacteriocin*, three-dimension graph.

The AFM planar imaging of* C. albicans* after* S. s bacteriocin* treatment was shown in Figure 4; it could be found that thallus was warped, overlapped, and deformed, the surface was rough and creased, and disc-like depressions appeared.

**Figure 5:**
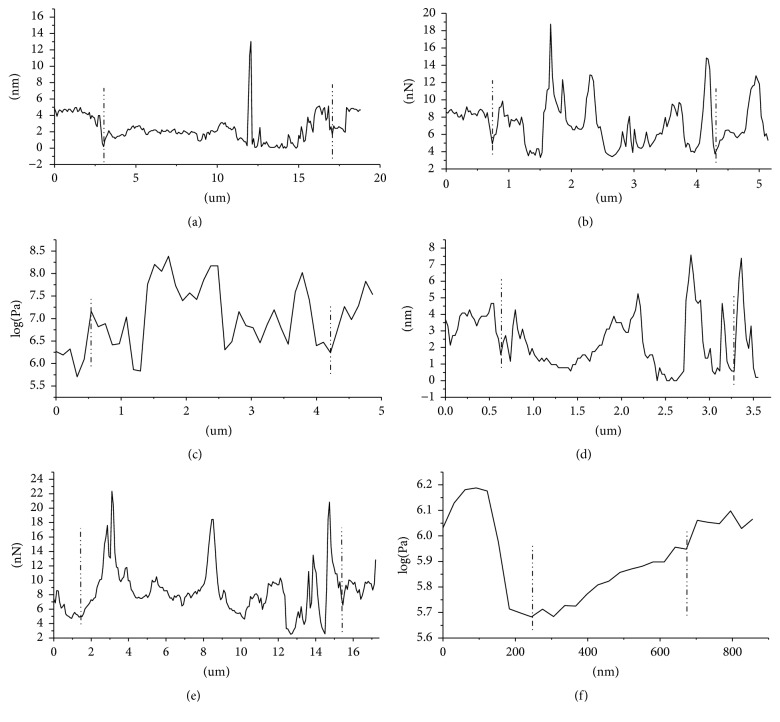
Effects of* S. s bacteriocin* on deformation, adhesion ability, and Young's modulus of* C. albicans*. (a) The graph of* C. albicans* deformation. (b) The graph of* C. albicans* adhesion. (c) The graph of* C. albicans* Young's modulus. (d) The graph of* C. albicans* deformation after* S. s bacteriocin* treatment. (e) The graph of* C. albicans* adhesion ability after* S. s bacteriocin* treatment. (f) The graph of* C. albicans* Young's modulus after* S. s bacteriocin* treatment.

After morphology imaging, deformation changes of* C. albicans* antagonized by* S. s bacteriocin* could be directly observed by AFM. Using AFM quantitative analysis, the value changes of deformation after* S. s bacteriocin *treatment could be directly measured (Figure 5(d)). The results showed that deformation of* C. albicans* changed significantly after* S. s bacteriocin *treatment (Table 3).

The adhesion ability of normal* C. albicans *was 7.35 ± 0.77 nN. The mean value was 5.69 ± 0.62 nN after* S. s bacteriocin *treatment (Table 4). It could be conformed that the adhesion ability of* C. albicans *was reduced* by S. s bacteriocin*, and the value was decreased along with the cup depth increasing (Figure 5(e)).

Young's modulus of normal* C. albicans *was 7.33 ± 1.29 Mpa. The mean value was 5.03 ± 0.81 Mpa after* S. s bacteriocin *treatment (Table 5). It could be conformed that Young's modulus of* C. albicans *was reduced* by S. s bacteriocin*, resulting in increasing of elasticity and deformation (Figure 5(f)).

## 4. Discussion 

*Candida albicans* was a kind of conditioned pathogen; it might induce disease through many ways, for example, recognizing the host by adhesive attraction and invading the host by transition from spore phase to hypha phase. The shape of* C. albicans* was plastic and variable; it could accommodate the internal environment of host by changing its shape. Adhesion was the first step of pathogenicity of* C. albicans*; it was the necessary condition for virulence factors to develop their pathopoiesis. Adhesion represented the ability of attachment to the surface of host or materials for bacteria; it had close relationships with virulence of pathogenic bacteria [18]. Young's modulus used to represent the size of cell elasticity and hardness reflects the ability of resistance to deformation; when Young's modulus was small, their elasticity was large; it is an important parameter for the measurement of cytomechanics; Young's modulus was closely related to the structure and function of pathogenic bacteria [19]. In the present, more and more research focused on cellular biomechanics [18]. It was reported that* S. s bacteriocin *could inhibit the growth of* C. albicans*; it could even change the cell shape of* C. albicans*, resulting in the increasing of cell membrane permeability [20]. However, the mechanism of the inhibitory effect of* S. s bacteriocin* had not been reported yet. In this study, the* S. s bacteriocin *was extracted by high speed centrifugation and cell disruption methods; the extracts were used for the treatment of* C. albicans *in spore phase and hypha phase; the spore and hypha were scanned by atomic force microscope (AFM), so the morphological and mechanical information of* C. albicans *before and after* S. s bacteriocin *treatment were obtained. It could provide some basis for illustrating the mechanical effects of* S. s bacteriocin* on* C. albicans*.

The result showed that the adhesion ability of normal hypha was 9.82 ± 0.39 nN, and the thallus was 7.35 ± 0.77 nN, the adhesion ability of hypha was greater than thallus (*p* < 0.05). It was reported that the formation of hypha was related to the increase of pathogenicity [21], but it was also shown that adhesion ability was the most important factor for the pathogenicity of* C. albicans*; it could represent the virulence of* C. albicans *[22]; the adhesion value was compared between hypha and thallus in this study; it was confirmed that the hypha pathogenicity was stronger than thallus in cytomechanics aspects. The mean value of Young's modulus of normal thallus was 7.33 ± 1.29 Mpa, and the hypha was 4.04 ± 0.76 Mpa; Young's modulus of hypha was less than thalli; namely, the elasticity of hypha was larger than thallus, so deformation was more likely to happen in the hypha rather than thallus. It was confirmed that* C. albicans* could accommodate the internal environment of host by changing its shape [23]. Fungi had not been clearly recognized in clinic; it was probably because morphological plasticity of hypha was much stronger than thallus, so it could accommodate to environmental changes more quickly and become more pathogenic. It was reported that the protein content from cell wall extracts of* C. albicans* in hypha phase was higher than spore phase, so when* C. albicans *was changed from spore phase to hypha phase, there will be new protein synthesis in cell wall or the increase of protein numbers [20]. In conclusion, it was confirmed in cytomechanics level that the pathogenicity of* C. albicans *in hypha phase was stronger than spore phase. The reasons were as follows. First, mucoprotein of* C. albicans *in hypha was higher than thallus or this kind of mucoprotein had acceptor which could combine to tissue and cells. Second, there was cell wall content in the thallus of* C. albicans*, while cell wall content might not be formed in hypha, or there were some differences in the cell wall structure and content between thallus and hypha. Third, ergosterol was an important component in fungal cell membrane; it could stabilize cell membrane structure and minimize the fluidity of cell membrane [24]. It was suspected that the content of ergosterol in the membrane of hypha was higher than thallus. The hypha could secrete the secreted aspartyl proteinases (Sap); there were 9 members in SAP gene family, namely, SAP 1~9; the substrate of their coding product had various kinds of proteins in the host; among them, SAP4~6 were expressed only in hypha phase [25, 26]. This might be a reason why pathogenicity of hypha was stronger than spore.

The shape of* C. albicans *had polytropy;* C. albicans *could coexist in many anatomic sites; there were many allosteric enzymes, 3 glycometabolism pathways, and 2 complete respiratory systems in* C. albicans*, so it had specific system to accommodate to the environment in every survival state. Thus it means that there was complexity in the therapy of candidiasis. If the adhesive attraction of* C. albicans *could be put into priority, blocking the first way of host infection, other complex pathogenic processes would not proceed, and the* C. albicans *infection could be cured. In this study,* C. albicans *in the gemmation stage was treated with* S. s bacteriocin* and their adhesion ability was measured. The result showed that the adhesion ability was decreased significantly after treatment, and their adhesion ability was decreasing with the rising of deformation value in the reaction value. The adhesion acceptor of* C. albicans *was tripeptide structure with Arginine-Glycine-Aspartic Acid (RGD), which could inhibit the adhesive attraction of* C. albicans* [27]. It could be suspected that there might be RGD structure in* S. s bacteriocin; *this structure could combine with attachment protein ligand of* C. albicans*, resulting in the decrease of adhesion ability of* C. albicans*. It was shown in previous study that* S. s bacteriocin* could induce the Ca^2+^ and Mg^2+^ leakage and increase the cell membrane permeability of* C. albicans* [7, 24]. Ca^2+^ and Mg^2+^ could increase the adhesion ability of fungi [28]. Combining the results in this study, it was suspected that* S. s bacteriocin* could reduce the adhesion ability of* C. albicans *by decreasing the amount of Ca^2+^ and Mg^2+^ in the cells. It was reported that* S. s bacteriocin* could reduce the hydrophobic interaction in the cell surface of* C. albicans, *while the hydrophobicity and adhesive attraction of* C. albicans* were positively correlated [7]. It was confirmed in this study that* S. s bacteriocin* could reduce the adhesion ability of* C. albicans*, but was this reduction accomplished by inhibiting the hydrophobicity of* C. albicans*? Or the hydrophobicity was affected by the adhesion reduction? Or they had their own function pathways? It needs further study.

Young's modulus was an important component which could represent the mechanics characteristics of the cell; it had positive correlation with cell hardness and rigidity, while it had negative correlation with cell elasticity. The outer layer of* C. albicans *was wrapped with rigid cell wall structure, when Young's modulus of* C. albicans *was measured by atomic force microscope; the cell surface, scanned by experimental probe, was the cell wall structure of* C. albicans*. In this study, Young's modulus of thallus was decreased significantly after* S. s bacteriocin *treatment, namely, the decrease of Young's modulus of cell wall, so the hardness of cell wall was decreased. The cell wall had the function of maintaining cell shape and increasing thallus mechanical property. In this study the shape of* C. albicans* was twisted and depressed after* S. s bacteriocin *treatment; the cell wall rigidity and hardness were decreased. Thus their function on maintaining cell shape and increasing thallus mechanical property had been lost. There were antigenic structures on the cell wall of* C. albicans*; the main antigenic component was mannoprotein, because the* S. s bacteriocin *had a polypeptide chain or glycoprotein, a kind of precursor protein, so they might combine to the mannoprotein in the cell walls and then change or destroy the structure of cell walls. It was suspected that the inhibition region of* S. s bacteriocin *on* C. albicans* might be the cell wall. While some studies showed that specific permease, a ATP enzyme in the cytoplasm membrane, was involved in the glycoprotein transportation of* C. albicans*, it could transport nutritive material and maintain a kind of positive proton movement [29]. The peptides transportation by* C. albicans *depended on this transport system. So it could be suspected that* S. s bacteriocin *could enter the inner cell of* C. albicans *through their active transport process and interfere in and destroy their metabolic processes of nutritive material or important ions, resulting in a series of changes, such as thallus permeability, adhesion ability, and cell wall hardness; finally the cells were induced to apoptosis.

In summary,* C. albicans* had numerous pathogenic pathways; study on antiblastic effect of* S. s bacteriocin *on* C. albicans *should be versatile. In this study, the effects of* S. s bacteriocin *on the adhesion ability and Young's modulus of* C. albicans *were studied in the aspects of cytomechanics; the results could provide some basics for the fungal mechanical characteristics which were induced by* S. s bacteriocin.*

## Figures and Tables

**Table 1:** Adhesion ability of *C. albicans* thalli and hyphae (nN).

Survey line	*A*1	*B*1	*C*1	*D*1	Mean value
Hypha	10.06	9.25	10.09	9.87	9.82 ± 0.39
Thalli	8.43	6.61	7.19	7.20	7.35 ± 0.77

*Note.* Paired-samples *T* test was applied, *t* = 4.171, and *p* = 0.025.

**Table 2:** Young's modulus of *C. albicans* thalli and hyphae (MPa).

Survey line	*A*2	*B*2	*C*2	*D*2	Mean value
Hypha	4.61	3.01	3.90	4.63	4.04 ± 0.76
Thalli	7.06	6.32	9.22	6.74	7.33 ± 1.29

*Note.* Paired-samples *T* test was applied, *t* = 4.580, and *p* = 0.020.

**Table 3:** Deformation changes of *C. albicans* after *S. s bacteriocin* treatment (nm).

Group	1	2	3	4	5	6	7	8	9	10
Control group	74.13	77.85	75.51	69.85	82.13	87.52	90.28	61.57	56.18	70.26
Test group	77.36	97.04	120.71	259.44	348.34	408.66	426.20	357.45	272.49	141.77

*Note*. Control group was *C. albicans*, test group was *C. albicans* treated with *S. s Bacteriocin*, paired-samples *T* test was applied, *t* = 4.273, and *p* = 0.002.

**Table 4:** Adhesion ability changes of *C. albicans* after *S. s bacteriocin* treatment (nN).

Group	1	2	3	4	5	6	7	8	9	10
Control group	5.62	6.02	5.61	6.25	5.33	5.85	5.38	5.36	5.39	5.62
Test group	4.59	3.83	3.86	2.91	2.49	2.34	3.24	3.67	4.45	5.33

*Note*. Control group was *C. albicans*, test group was *C. albicans* treated with *S. s bacteriocin,* paired-samples *T* test was applied, *t* = −5.923, and *p* < 0.001.

**Table 5:** Young's modulus changes of *C. albicans* after *S. s bacteriocin* treatment (MPa).

Group	1	2	3	4	5	6	7	8	9	10
Control group	7.63	7.58	7.29	7.37	7.41	7.55	7.72	7.66	7.39	7.55
Test group	5.96	5.37	4.69	4.34	3.99	4.27	4.57	4.88	5.66	6.64

*Note.* Control group was *C. albicans*, test group was *C. albicans* treated with *S. s bacteriocin*, paired-samples *T* test was applied, *t* = −9.490, and *p* < 0.001.
